# Integration of Cardiac Actin Mutants Causing Hypertrophic (p.A295S) and Dilated Cardiomyopathy (p.R312H and p.E361G) into Cellular Structures

**DOI:** 10.3390/antiox10071082

**Published:** 2021-07-05

**Authors:** Constanze Erdmann, Roua Hassoun, Sebastian Schmitt, Carlos Kikuti, Anne Houdusse, Antonina J. Mazur, Andreas Mügge, Nazha Hamdani, Matthias Geyer, Kornelia Jaquet, Hans Georg Mannherz

**Affiliations:** 1 Department of Anatomy and Molecular Embryology, Medical Faculty, Ruhr-University Bochum, D-44780 Bochum, Germany; Constanze.Erdmann@rub.de; 2 Institut für Forschung und Lehre (IFL), Molecular and Experimental Cardiology, Ruhr University Bochum, D-44780 Bochum, Germany; Roua.Hassoun@rub.de (R.H.); andreas.muegge@ruhr-uni-bochum.de (A.M.); nazha.hamdani@ruhr-uni-bochum.de (N.H.); kornelia.jaquet@rub.de (K.J.); 3 Department of Cardiology, St. Josef-Hospital and Bergmannsheil, Ruhr University Bochum, D-44780 Bochum, Germany; 4 Institute of Structural Biology, University of Bonn, D-53127 Bonn, Germany; sebastian.schmitt@gmx.at (S.S.); Matthias.Geyer@uni-bonn.de (M.G.); 5 Institut Curie, Structural Motility Team, F-75005 Paris, France; carlos.kikuti@curie.fr (C.K.); Anne.Houdusse@curie.fe (A.H.); 6 Department of Cell Pathology, Faculty of Biotechnology, University of Wroclaw, Pl-50-383 Wroclaw, Poland; antonina.mazur@uwr.edu.pl

**Keywords:** ATPase, Arp2/3 complex, cardiac actin, cardiomyopathies, MICAL

## Abstract

The human mutant cardiac α-actins p.A295S or p.R312H and p.E361G, correlated with hypertrophic or dilated cardiomyopathy, respectively, were expressed by the *baculovirus/Sf21* insect cell system and purified to homogeneity. The purified cardiac actins maintained their native state but showed differences in Ca^2+^-sensitivity to stimulate the myosin-subfragment1 ATPase. Here we analyzed the interactions of these c-actins with actin-binding and -modifying proteins implicated in cardiomyocyte differentiation. We demonstrate that Arp2/3 complex and the formin mDia3 stimulated the polymerization rate and extent of the c-actins, albeit to different degrees. In addition, we tested the effect of the MICAL-1 monooxygenase, which modifies the supramolecular actin organization during development and adaptive processes. MICAL-1 oxidized these c-actin variants and induced their de-polymerization, albeit at different rates. Transfection experiments using MDCK cells demonstrated the preferable incorporation of wild type and p.A295S c-actins into their microfilament system but of p.R312H and p.E361G actins into the submembranous actin network. Transduction of neonatal rat cardiomyocytes with adenoviral constructs coding HA-tagged c-actin variants showed their incorporation into microfilaments after one day in culture and thereafter into thin filaments of nascent sarcomeric structures at their plus ends (Z-lines) except the p.E361G mutant, which preferentially incorporated at the minus ends.

## 1. Introduction

Hereditary cardiomyopathies (CM) are mainly caused by mutations in genes encoding sarcomeric [1,2,3] or cytoskeletal proteins [4,5,6,7]. Hypertrophic cardiomyopathy (HCM) is characterized by a thickening of the left ventricular wall (often eccentric) accompanied by increased stiffness of the cardiac muscle resulting in reduced ventricular filling during diastole. Dilated cardiomyopathy (DCM) is characterized by enlarged left and/or right ventricles with a reduced ejection volume during systole. HCM has a genetic background in about 90% of all cases with a prevalence based on new estimates of 1:200 in the general population; DCM is in only 20% genetically caused with a prevalence of 1:250 [8]. The remaining 80% DCM cases develop as complications of bacterial or viral infections, adverse lifestyle, alcoholism, drug abuse, or chemotherapy [3,4]. For the year 2015, the global burden of disease study estimated the total prevalence of cardiomyopathies to be 2.5 million [9]. Single point (missense) mutations appear responsible for the development of cardiomyopathy. So far, about 1400 different missense mutations have been identified in sarcomeric or cytoskeletal proteins causing HCM or DCM preferentially, respectively [1,2,3,4,5,6,7]. The most frequently affected proteins causing HCM are the cardiac ß-myosin and the myosin-binding protein C (cMyBP-C). Mutations of the cardiac α-actin gene (ACTC) were also identified in HCM and DCM patients; however, they occur with an incidence of only 4–6% of patients suffering from familial cardiomyopathy [7,10,11,12,13,14,15]. Presently, 14 different missense mutations causing HCM and two DCM have been identified in cardiac (c-)actin [7,15]. Thus, mutations of c-actin causing different cardiomyopathy phenotypes occur in the same protein molecule.

Provided that a missense mutation does not impair the native state of the affected c-actin, pathological consequences can only arise by alterations of its mode of interaction with other regulatory proteins during cardiomyocyte development or of the sarcomere [14,15]. Therefore, it appears plausible that the localization of the mutated residue within a surface area responsible for a specific protein-protein interaction, like, for instance, myosin heads, determines the type of cardiomyopathy [16]. Generally, hyper- or hypocontractility have been linked to the development of HCM or DCM, respectively [17]. Therefore, HCM mutations may mainly be gain-of-function mutations, whereas DCM mutations may lead to loss-of-functions. Furthermore, mutations causing cardiomyopathies act in a dominant-negative manner and are, therefore, in most cases single-allelic. The mutated protein is assumed to override in a poisonous manner the counterpart of the healthy allele [3,18] though for many cases the mechanisms, by which a given missense mutation leads to a HCM or DCM phenotype are not fully understood.

Recently, oxidative stress has been shown to significantly contribute to the phenotypic development of cardiomyopathies and heart failure [19]. Even familial cardiomyopathies (HCM and DCM) are accompanied by an increase in oxidative stress that may promote the establishment of the cardiomyopathy phenotype [20,21,22]. In this context, proteins of the MICAL family may be of high relevance because they are involved in the differentiation and adaptive processes of many cell types by inducing rapid disassembly of existing actin filaments [23,24,25]. MICAL-1 achieves F-actin de-polymerization by oxidation of methionines 44 and 47 of actin leading to reduced polymerizability [23]. For instance, in neuronal cells, MICAL proteins execute repulsive signals from different external cues regulating axonal guidance [19]. Together with methionine sulfoxide reductase B (MSRB), which reduces the oxidation of the methionines restoring actin function, MICAL-1 might participate in the dynamic control of the polymerization state of the actins present in cardiomyocytes thereby regulating their incorporation into micro- and thin filaments or its turnover in adult cardiomyocytes.

Previously different aspects of the consequences of c-actin mutations have been analyzed in a number of different investigations [10,11,12,13,14,15,16,26]. In earlier reports, we described the expression, purification, and biochemical properties of c-actin variants [27]. So far, we analyzed three HCM causing c-actin mutants (p.Y166S, p.M305L, and p.A295S) and two DCM (p.R312H and p.E361G) causing mutations using biochemical procedures we characterized their properties and searched for differences in their mode of interaction with other sarcomeric proteins.

Here we further investigated the cell biological properties of the p.A295S, p.R312H, and p.E361G mutants by studying their incorporation into cytoskeletal elements of established cell lines and into sarcomeric thin filaments of neonatal rat cardiomyocytes. Furthermore, after recombinant expression and purification as native and tag-free proteins, we analyzed their interaction with proteins that may play a role during cardiomyocyte development-like proteins that promote the nucleation and polymerization of actin. Thus, we investigated the effects of the polymerization nucleators Arp2/3 complex and mDia3 on their rates of polymerization. Since oxidative stress may contribute to the development of cardiomyopathies, we tested the response of the c-actin mutants to the F-actin specific methionine monooxygenase MICAL-1 (molecule interacting with CasL-1). The occurrence of MICAL-1 in muscle cells has been demonstrated for *Drosophila* myocytes [28] but not yet for mammalian cardiomyocytes. Therefore we searched for its presence in cardiomyocytes isolated from adult rats. In addition, we determined the rates of oxidation of the cardiac actin variants by following the NADPH consumption by the N-terminal fragment of MICAL-1 and the concomitant de-polymerization of the modified c-actin variants.

## 2. Materials and Methods

### 2.1. Materials

#### 2.1.1. Antibodies

Monoclonal mouse anti-skeletal α-actin (clone AC-1-20.4.), anti-ß-actin (clone AC15), mouse anti-sarcomeric α-actinin, rabbit anti-all actins (clone C11) antibodies were obtained from Sigma-Aldrich (Munich, Germany). The monoclonal mouse anti-MICAL-1 antibody was purchased from Abcam (Cambridge, UK). Donkey anti-mouse Alexa Fluor^®^ 488 or 568 and anti-rabbit Alexa Fluor^®^ 488 or 568 antibodies were from Molecular Probes (Eugene, OR, USA). Goat anti-HA (clone Y-11) antibody was obtained from Santa Cruz Biotechnology (Dallas, TX, USA); mouse anti-cardiac α-actin was purchased from Progen Biotechnik GmbH (Heidelberg, Germany). Monoclonal anti-myomesin (clone B4) antibody (Grove et al., 1984) was a kind gift from Dr. E. Ehler (King’s College London, London, UK). All primary and secondary antibodies were employed in a 1:200 and 1:500 dilution, respectively.

#### 2.1.2. Clones

The pcDNA3.1/NT-GFP-TOPO^®^-WT-α-cardiac actin and the mutants p.A295S, p.R312K, and p.E361G were donated from Dr. Cora-Ann Schoenenberger (University Basel, Switzerland). The p.R312H mutant was generated by site-directed mutagenesis from the p.R312K variant. These c-α-actin containing plasmids served as templates for cloning the c-α-actin variants into the p3xHA-C1 plasmid. The p3xHA-C1 plasmid was a kind gift from Dr. T. Engel (Leibniz-Institut für Arteriosklerosis, Münster University, Germany), who deleted cDNA of EGFP from the pEGFP-C1 plasmid (Clontech) and instead cloned into this plasmid the cDNA of a hemagglutinin-tag (HA) repeated three times. The primers used for amplifying the actin cDNAs were as follows: 5′-GTTATGTGTGACGACGAGGAGACC-3′ and 5′-ATTGCCCTTTTAGAAGCATTTGCG-3′. PCR inserts were cloned into p3xHA-C1 using XbaI and XhoI sites.

The deletion construct of human gelsolin G4-6 was kindly supplied by Dr. A.G. Weeds (MRC, Cambidge, UK) and subcloned from shuttle vector pKN172 into the cold-shock expression plasmid pCOLD II (Takara Bio Inc., Kusatsu, Japan) using the restriction sites for *Bam*HI and *Hind*III enzymes obtained from Fermentas (Vilnius, Lithuania). The pCOLD II plasmid provides a His-Tag sequence for affinity chromatography, which was fused to the N-terminus of G4-6, which was used to affinity-purify the c-α-actin variants [27]. Arp2/3 complex isolated from *Acanthameba castellani* was kindly supplied by Prof. M. Barber (San Francisco, CA, USA) and mDia3-FH2 by Prof. Alfred Wittinghofer (MPI, Dortmund, Germany).

#### 2.1.3. Protein Expression and Purification

Cardiac bovine α-actin was prepared from dried acetone powder [29]. Human cardiac muscle wild-type α-actin and its mutants were expressed in the baculovirus/Sf21-system as detailed in [7,27,30]. Briefly, after centrifugation of the SF21 homogenate, the supernatant was incubated with His-tagged gelsolin-G4-6 in the presence of 1 mM CaCl_2_ and thereafter incubated with Ni^2+^-NTA agarose beads (Invitrogen, Schwerte, Germany). The affinity bound c-actin variants were subsequently eluted by incubation with 5 mM EGTA, dialyzed against 5 mM HEPES-OH, pH 7.4, 0.1 mM CaCl_2_, 0.5 mM NaN_3_, and 0.2 mM ATP. After dialysis, the c-actins were supplemented with 5% sucrose and stored at −80 °C until use. The bacterial expression of the *N*-terminal domain of MICAL-1 containing the monooxygenase activity and its purification has been described previously [30].

#### 2.1.4. Cardiac Actin Oxidation by MICAL-1

Methionine oxidation of the cardiac actin variants by MICAL-1 was determined by the decrease in absorbance at 340 nm of NADPH at 25 °C using either a Beckman D800 or a Specord200 photometer from Analytik Jena (Jena, Germany). Cardiac actins polymerized by 2 mM MgCl_2_ and 50 mM KCl at a final concentration of 1 µM were treated with 0.5 µg of the monooxygenase activity containing *N*-terminal fragment of MICAL-1 in the presence of 20 or 40 µM NADPH [31].

#### 2.1.5. Gel Electrophoresis

Protein concentrations were determined by á colorimetric assay [32]. Polyacrylamide gel electrophoresis in the presence of SDS (SDS-PAGE) was performed as described in [33].

#### 2.1.6. Actin Polymerization Assays

Actin polymerization rates were determined by the increase in fluorescence caused by the incorporation of pyrene-labeled actin into actin filaments [34,35]. Pyrene-labeled actin was pre-cleared by dialysis against G-buffer (10 mM Tris-HCl, pH 8.0, 0.2 mM CaCl_2_, 7 mM β-mercaptoethanol, 1 mM ATP) and centrifugation at 100,000× *g* for 30 min. In these tests, we used pyrene-labeled skeletal muscle actin that was added to the c-actins at a ratio of 20:1 (0.25% to 5% of the c-α-actin). Since pyrene-labeled skeletal-actin on its own at 0.25 µM did not show significant polymerization, i.e., increase in fluorescence. Therefore, we assume that the increase in fluorescence observed after mixing it with globular c-α-actins in G-buffer was solely due to the polymerization of the c-α-actins. Polymerization was induced by the addition of 2 mM MgCl_2_ and 0.1 M KCl. The increase in pyrene-fluorescence was monitored using a Schimadzu RF5001PC or a Perkin-Elmer spectrofluorometer with excitation and emission wavelength settings of 365 nm and 385 nm, respectively [34,35]. De-polymerization of F-c-actins induced by MICAL-1 was determined similarly with 5 µM F-c-actin variant supplemented with pyrene-labeled skeletal muscle actin by measuring the decrease in fluorescence after addition of NADPH [24,35].

### 2.2. Cell Culture and Immunohistological Procedures

#### 2.2.1. Culture Cells

Hela and MDCK (Marvin-Darby canine kidney) cells were propagated in DMEM medium containing 0.5% glucose, 1% penicillin/streptomycin, 1% glutamine, 0.5% sodium pyruvate, and 10% fetal calf serum. Cells were cultured in 25 cm^2^ flasks (Falcon^®^, Becton Dickinson GmbH, Heidelberg, Germany) at 37 °C in 5% CO_2_ and 90% humidified air and split weekly, using 0.25% trypsin/0.05% EDTA solution. MDCK cells were kindly supplied by Prof. Anna Starzinski-Powitz (Frankfurt, Germany). Cardiomyocytes from 1–5 days old rats (neonatal rat cardiomyocytes; NRCs) and from adult rats (ARCs) were isolated following the protocols described in [27,36]. ARCs were kindly provided by Nicole Rabev (Bochum, Germany).

#### 2.2.2. Cell Transfection

The cells were seeded on glass coverslips in 6-well plates (3 × 10^5^ cells/well) and transfected with the help of MATra-A reagent (Iba, Munich, Germany) with 3 µg of DNA encoding either for EGFP-actins or HA-actins as detailed [37]. For Western blot analysis, the cells were seeded in 6 cm plastic Petri dishes and transfected with 5 µg DNA. Twenty-four or forty-eight hours after transfection, the cells on coverslips were fixed with 4% formaldehyde (FA) or harvested in Petri dishes in lysis buffer using a rubber policeman.

#### 2.2.3. Generation of Recombinant Adenoviruses

For the generation of recombinant adenoviruses (Ad) the AdEasy™ kit (Qbiogene) was applied [7,27,38]. DNA sequences encoding WT- and the mutants A295S-, R312H-, and E361G- c-α-actins fused at the *N*-terminus to an HA-tag were amplified by PCR with the primers: 5′ATCATGGATTACCCATACGATGTTC-3′ and 5′-ATCGCCCTTTTAGAAGCATTTGCG-3′. p3xHA-C1 plasmids encoding wt and the mutant cardiac α-actins served as templates. The *EcoR*V site was used to clone PCR inserts into pAdTrack-CMV shuttle plasmid. Electro-competent bacteria *E.coli* BJ5183 were simultaneously transformed with the shuttle plasmid linearized with the help of *Pme*I and adenoviral AdEasy-1 DNA backbone. Following homologous recombination in bacteria, clones were screened by restriction with the *Pac*I enzyme that, in the case of positive clones, resulted in two 33 kb and 4.5 kb fragments. Lipofectamine™ (Invitrogen) reagent was used to transfect HEK293 cells with linearized pAdEasy-1 construct encoding wild type and the mutant cardiac α-actins. Since pAdEasy-1 lacks E1 and E3 genes critical for the successful packaging of adenoviruses, it was crucial to generate adenoviral particles in HEK293 cells, which contain these two genes. The adenoviral DNA encoded EGFP, additionally enabling tracking the generation of viral particles. After two to three weeks, the cells were lysed, liberating viral particles. HEK293 cells were twice re-infected with recombinant adenoviruses in order to obtain higher amounts of viral particles. For more details concerning the structure of recombinant adenoviral DNA and the steps of recombinant adenoviruses generation, see [7,27,38]. The correctness of DNA constructs was verified by sequencing using a DNAstar Lasergene software (DNASTAR Inc., Madison, WI, USA).

#### 2.2.4. Transduction of NRCs with Recombinant Adenoviruses

NRCs were infected with 20 µL of adenoviruses added to 2 mL of medium following the procedure described [27]. Seventy-two hours after infection, the cells were fixed with warm (37 °C) 4% formaldehyde (FA) for immunocytochemistry. For controls, cells were infected with only EGFP-encoding viruses.

#### 2.2.5. Confocal Microscopy

Control cells, transfected cells, and those infected with adenoviruses were fixed with warm (37 °C) 4% formaldehyde for 20 min at RT and permeabilized with 0.1% Triton X-100 in PBS for 6 min. For staining with anti-cardiac α-actin monoclonal antibody, we additionally fixed the cells with ice-cold methanol for 6 min at 4 °C. After fixation, the coverslips or plastic dishes were blocked for 30 min with 3% BSA in PBS. All antibodies were diluted in 3% BSA in PBS. The cells were immunostained either with goat anti-HA IgGs, or with monoclonal antibodies directed against anti-β-actin, anti-c-α-actin, anti-sarcomeric α-actinin, and anti-myomesin. The secondary IgGs were conjugated either with Alexa Fluor^®^ 488 or Alexa Fluor^®^ 568. In the case of double immunostaining, when the goat anti-HA antibody was applied, donkey anti-mouse IgGs were used in order to avoid cross-reactivity. F-actin was visualized by staining with TRITC-conjugated phalloidin (Sigma-Aldrich, St. Louis, MO, USA). The nuclei were visualized with the help of Hoechst 33342 (Riedel-de-Haen, Seelze, Germany). The coverslips or plastic dishes were washed several times with PBS for 5 min. After all incubations and washing steps, the cells were mounted in DAKO cytomatic fluorescent mounting medium. Immunofluorescence microscopy was performed using a Zeiss LSM 800 laser-scanning microscope (Jena, Germany). For documentation, at least 5 cells were photographed from three independent experiments, and a representative image is presented. Co-localization analysis was performed by using ZEN 2007 software (Carl Zeiss Vision GmbH, Göttingen, Germany) and confirmed when the Pearson’s correlation coefficient was >0.3.

## 3. Results

### 3.1. Expression and Purification of the Cardiac α-Actin Variants

**Figure 1:**
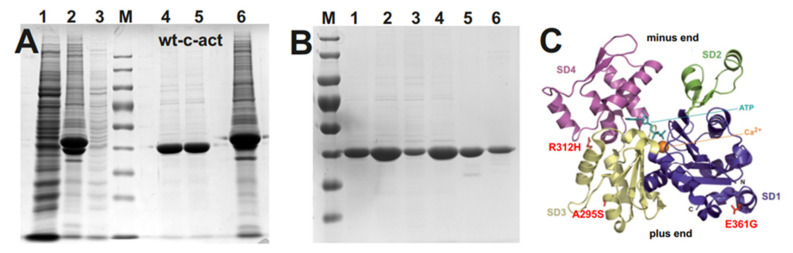
Purification of cardiac actins after expression by the *Baculovirus/Sf21* system. (**A**) SDS-PAGE (12.5%) of examples of the purification steps of cardiac actin variants as shown for wt c-actin. Lanes: (1) crude extract of Sf21 cells before addition of His-G4-6; (2) material bound to NiNTA beads; (3) supernatant of NiNTA beads washing step with calcium-containing Hepes-OH buffer, pH 7.4; (4) actin eluted after addition of Hepes buffer containing 5 mM EGTA; (5) cardiac bovine actin as comparison; (6) NiNTA beads after elution of actin showing remaining His-tagged G4-6. (**B**) SDS-PAGE of the purified cardiac actin variants used in this study. Lanes: (1) wt; (2) p.A295S; (3) p.R312H; (4) p.E361G; (5) skeletal muscle actin; (6) bovine cardiac actin. Actins shown in lanes (5) and (6) were conventionally prepared from acetone powders. (**C**) Gives the localization of the mutated residues within the actin molecule [modified from 42].

Expression of the c-α-actins (wt plus the mutants) was achieved in *Sf21* insect cells by using the *Baculovirus* system and purified after affinity binding to gelsolin-G4-6 as reported previously [7,18] as shown for wt c-actin in Figure 1. The isolated c-actin variants showed only one main band after SDS-PAGE (Figure 1B) and were previously shown to be cardiac α-actin by immunoblotting using an anti-cardiac α-actin antibody [7,18]. Cardiac actin was also conventionally purified from acetone powder obtained from bovine hearts. After SDS-PAGE its migration behavior and reactivity in Western blots against anti-cardiac α-actin mAB were found identical to recombinant wt c-α-actin [7,18]. The localization of the mutated residues is indicated in the 3D model of actin (Figure 1C).

### 3.2. Polymerization Behaviour of Recombinant c-α-Actins in the Presence of Nucleators

**Figure 2:**
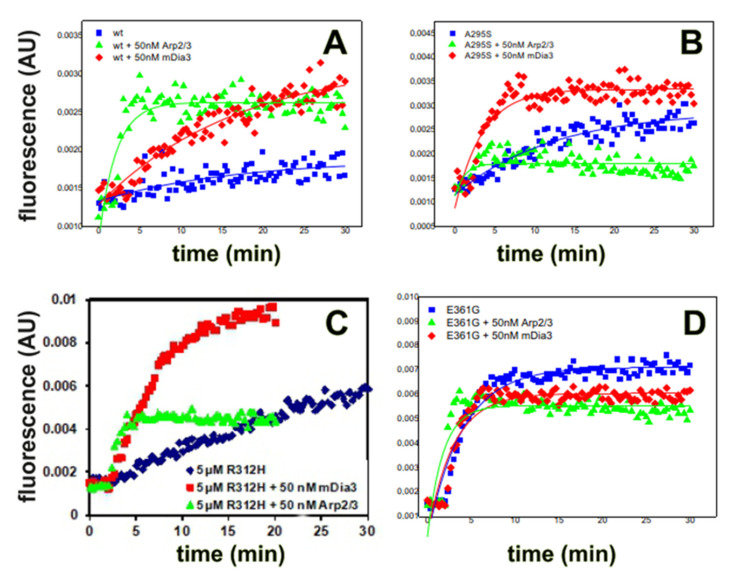
Polymerization of the purified c-actins in the presence of nucleators. (**A**–**D**) Influence of nucleators on the rates of polymerization of c-actins. (**A**) Recombinant wt-c-actin, (**B**) p.A295S, (**C**) p.R312H, and (**D**) p.E361G. All c-actins at 5 µM and in the absence of presence of either 50 nM Arp2/3 complex or 50 nM mDia3-FH2 (for details, see text). Representative experiments from three for every c-actin variant.

During cardiomyocyte differentiation and sarcomerogenesis, a number of different actin-binding proteins are involved in the processes of microfilament formation and transformation into sarcomeres. Indeed, recent data have shown the presence of actin polymerization nucleating factors (nucleators) in cardiac muscle [37]. Therefore, we analyzed the polymerization behavior of the c-α-actin variants in the presence of the nucleators Arp2/3 complex and the active FH2-domain of the formin mDia3, which induce either actin filament networks or straight filaments, respectively [38]. In these experiments, the nucleators were added to the c-actins at a 100:1 ratio before initiating polymerization by the addition of 2 mM MgCl_2_. The data showed that both nucleators stimulated the polymerization rate of all c-α-actin variants, albeit to different extents (Figure 2). The calculated half times of their polymerization are compiled in Table 1. The rate of wt c-actin was stimulated by the Arp2/3 complex more strongly than by mDia3 (Figure 2A, Table 1). A similar stimulation behavior on the polymerization was observed for the mutant c-actins (Figure 2; Table 1), though their polymerization rates were affected to different extents. The HCM p.A295S mutant was clearly stimulated by mDia3-FH2, but only minimally by Arp2/3 complex (Figure 2B). In contrast, the DCM mutant p.R312H was stimulated by both nucleators albeit more strongly by mDia3-FH2 (Figure 2C, Table 1). In contrast, Arp2/3 complex had only a small stimulating effect on the rate of polymerization of the p.E316G mutant, whereas mDia3-FH2 appeared to hardly affect its polymerization behavior (Figure 2D, Table 1). In summary, these data show differences in the polymerization behavior of the analyzed c-α-actin variants and its modulation by these nucleating proteins that might have functional consequences for their thin filament incorporation during cardiomyocyte development.

### 3.3. Oxidation of c-Actin Variants by MICAL-1

**Figure 3:**
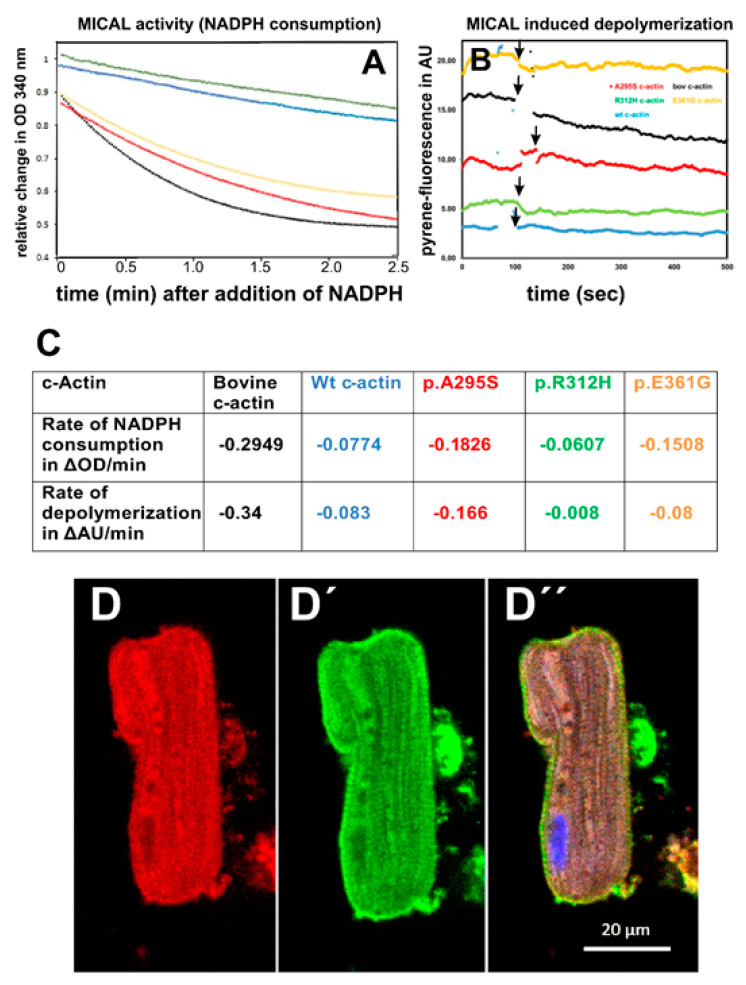
Influence of the monooxidase MICAL-1 on cardiac actin variants. (**A**) Rates of oxidation of c-actin variants by MICAL-1 as measured by substrate NADPH consumption determined optically at 340 nm (for details, see text). Cardiac actins at 1 µM were with 0.5 µg of the N-terminal domain of MICAL-1 in 5 mM HEPES buffer, pH 7.4, supplemented with 2 mM MgCl_2_ and 20 µM NADPH. Bovine c-actin (^__^); wt recombinant c-actin (^__^); p.A295S (^__^); p.R312H) (^__^); p.E361G (^__^). (**B**) Oxidation of the F-c-actin variants by MICAL-1 induced their de-polymerization. Representative experiments from three for every c-actin variant. (**C**) Table of the rates determined for the oxidation of the c-actins (**A**) and their de-polymerization (**B**). (**D**) Double immunostaining of ARCs with a mouse anti-MICA-L1 antibody and (**D’**) with a rabbit anti-actin antibody. (**D”**) Merged image together with nuclear Hoechst 33342 staining. Bar corresponds to 20 µm.

Recent evidence indicated that the main substrate of the MICAL-family monooxygenases is filamentous actin, which depolymerizes after oxidation of two methionines (Met 44 and 47 [24]). MICAL proteins are involved in many developmental and differentiation processes by stimulating the fragmentation of existing actin filaments. The c-actin variants were treated by the monooxygenase activity containing the N-terminal domain of MICAL-1 as detailed in Materials and Methods. Different rates of oxidation of the polymerized c-actins were observed (Figure 3A). Fast rates of oxidation by MICAL-1 were observed for bovine and the p.A295S and p.E361G mutants, whereas slower rates of oxidation were obtained for wt recombinant and the p.R312H mutant (Figure 3A,C).

Next, we tested the ability of MICAL-1 to induce the de-polymerization of the F-c-actin variants by following the decrease in the fluorescence of pyrene-labeled F-actin, which had been co-polymerized to 5% with the c-actins (Figure 3B). The data obtained showed different rates of de-polymerization being fastest for bovine, followed by p.A295S and wt-c-actin, but considerably slower for both DCM mutants, the p.R312H and p-E361G c-actins (Figure 3B,C), in partial agreement with the rates of modification.

In addition, we verified the presence of MICAL-1 by immunostaining using the monoclonal anti-MICAL-1 antibody at a 1:200 dilution in adult rat cardiomyocytes (ARCs) co-stained with an anti-actin antibody (Figure 3D). Figure 3D shows the longitudinally oriented myofilaments showing clear cross-striation by the anti-actin staining Figure 3D’). Anti-MICAL-1 staining showed clear staining along the myofilaments with a partial cross-striation, though a clear, direct co-localization with actin was not obvious (Figure 3D”). Nevertheless, MICAL-1 appeared to reside in close proximity to the actin-containing thin filaments.

### 3.4. Transfection of Established Cell Lines with Cardiac Actin Variants

**Figure 4:**
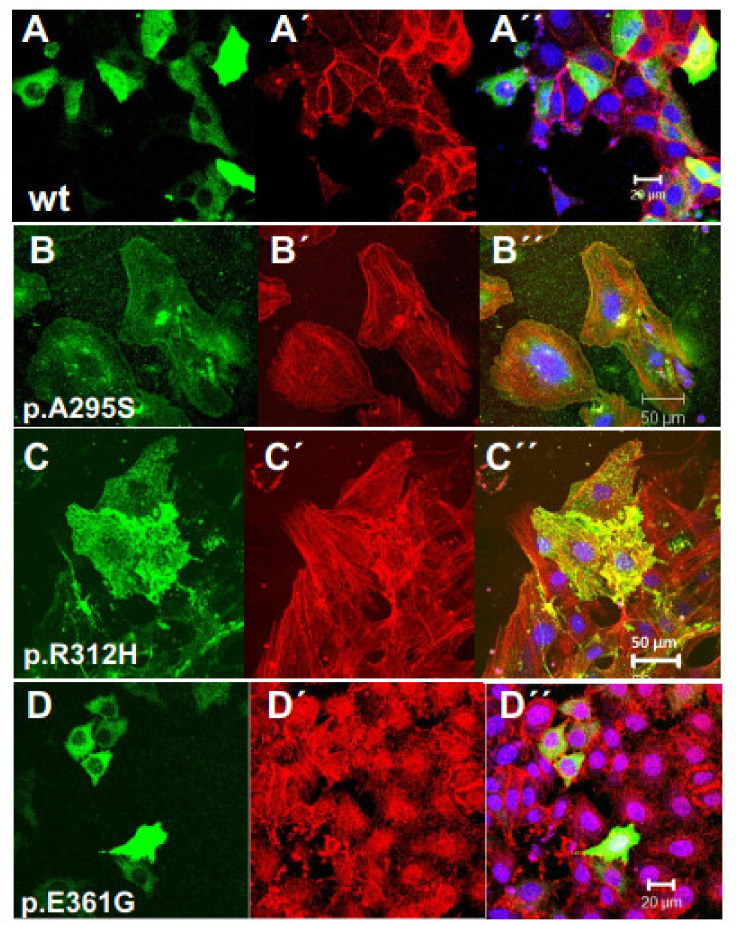
Transfection of Hela cells with EGFP-tagged c-actin variants. (**A**–**D**) EGFP-fluorescence, (**A’**–**D’**) TRITC-phalloidin staining, and (**A”**–**D”**) merged images together with Hoechst 33342 staining. (**A**) Wild type (wt) c-actin; (**B**) p.A295S mutant; (**C**) p.R312H mutant; (**D**) p.E361G mutant. For details, see text. Bars: (**A**,**D**): 20 µm; (**B**,**C**): 50 µm.

The physiological or cell biological effects of these cardiac actin variants were tested by transfection experiments into established cell lines like Hela and MDCK cells. First, we transfected Hela cells with N-terminally EGFP-tagged c-actins (Figure 4). After 24 h, the cells were fixed, counterstained with TRITC-phalloidin and Hoechst 33342 [37], and examined by confocal microscopy in order to identify the localization of the c-actin variants in relation to the microfilament system. The data indicated that wt c-actin and the p.A295S (Figure 4A,B) and partly also the p.R312H mutant (Figure 4C) integrated into the stress fibers of the microfilament system. In contrast, the p.E361G mutant and also to a lesser degree the p.R312H mutant gave a more punctuate staining (Figure 4D”) either within or outside the endogenous microfilaments (p.R312H see Figure 4C”) suggesting a localization distinct from the long stress fibers.

**Figure 5:**
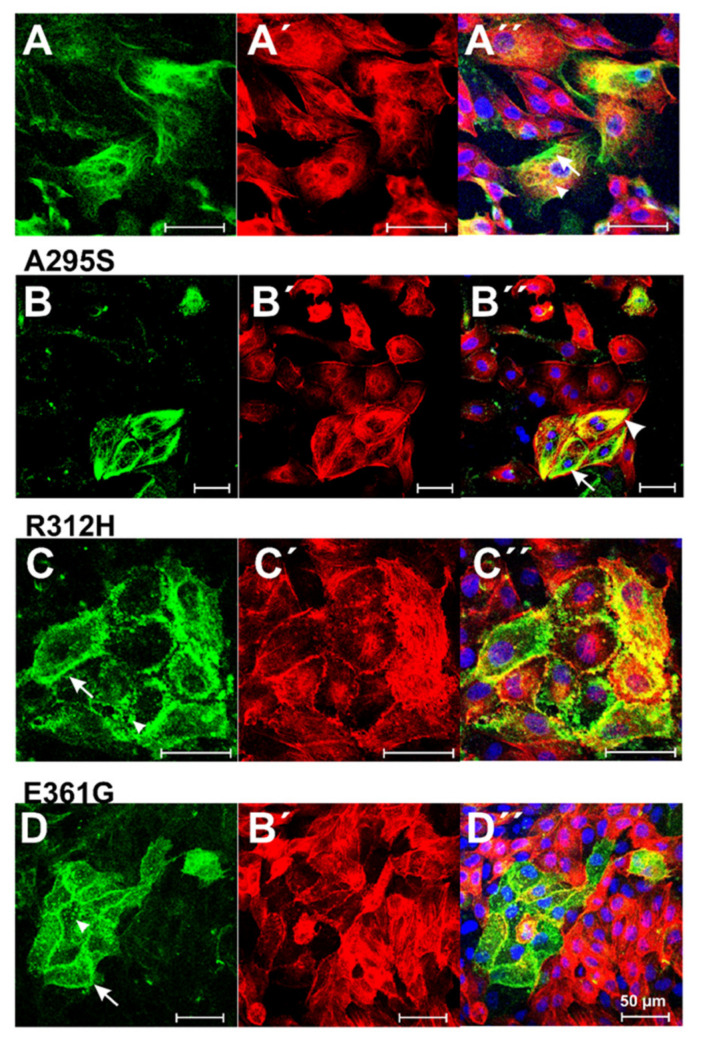
Transfection of MDCK cells with HA-tagged c-actin variants. The MDCK cells were transfected with vectors containing HA-tagged c-actin variants (for details, see Materials and Methods). Subsequently, the cells were immunostained with anti-HA and counterstained with TRITC-phalloidin. (**A**) wt c-actin stained with anti-HA (green); (**A’**) TRITC-phalloidin staining;. (**B**–**B’**) p.A295S mutant; (**C**–**C’**) p.R312H mutant; and (**D**–**D’**) p.E361G mutant; all with identical staining procedure. Note for WT and p.A295S mutant c-actins the preferential incorporation in the stress fiber system, whereas p.R312H and p.E361G mutants showed a more circumferential anti-HA staining suggesting preferred incorporation into short cortical actin filaments. (**A”**–**D”**) give merged images including nuclear stain by Hoechst 33342 not shown separatly. Bars: 20 µm.

When using EGFP-tagged c-actins for transfection experiments, we noticed largely different extents of expression. In particular, their overexpression made it difficult to reliably identify their incorporation into the endogenous microfilament system. The reasons for these difficulties might be the large size of the *N*-terminally attached EGFP (green-fluorescent-protein: about 27 kDa) that might indeed impair integration into actin filaments. Therefore we subsequently employed c-α-actins that were *N*-terminally tagged with a threefold hemagglutinin (HA-) extension (molecular mass about 300 daltons; see Materials and Methods). Furthermore, we used the polarized MDCK cells, which have an apical terminal web composed of intermingled actin filaments connected to junctional complexes of the adhesion belt that are clearly distinct from the stress fiber system within the cell body. After transfection, these cells were fixed, immunostained with anti-HA antibody, TRITC-phalloidin, and Hoechst 33342, and examined by confocal microscopy. The results obtained indicated that all c-α-actin variants incorporated into the cellular cytoskeleton of the MDCK cells. The data shown in Figure 5 demonstrate that wt and p.A295S (HCM) mutant c-α-actin preferentially incorporated into stress fibers forming either filamentous structures composed of apparently only the transfected c-α-actins as indicated by anti-HA staining (green; arrows) or co-polymerizing with the endogenous actin (yellow arrowheads) in the merged images (Figure 5A”,B’). In contrast, the p.R312H and p.E361G mutants did not significantly form stress fibers but appeared to incorporate into apical structures of the polarized MDCK cells like the sub-membranous actin belt underneath presumed junctional contacts (arrowheads; Figure 5C,D) and microvillar extensions (arrows in Figure).

### 3.5. Infection of Rat Neonatal Cardiomyocytes with the Cardiac Actin Variants

**Figure 6:**
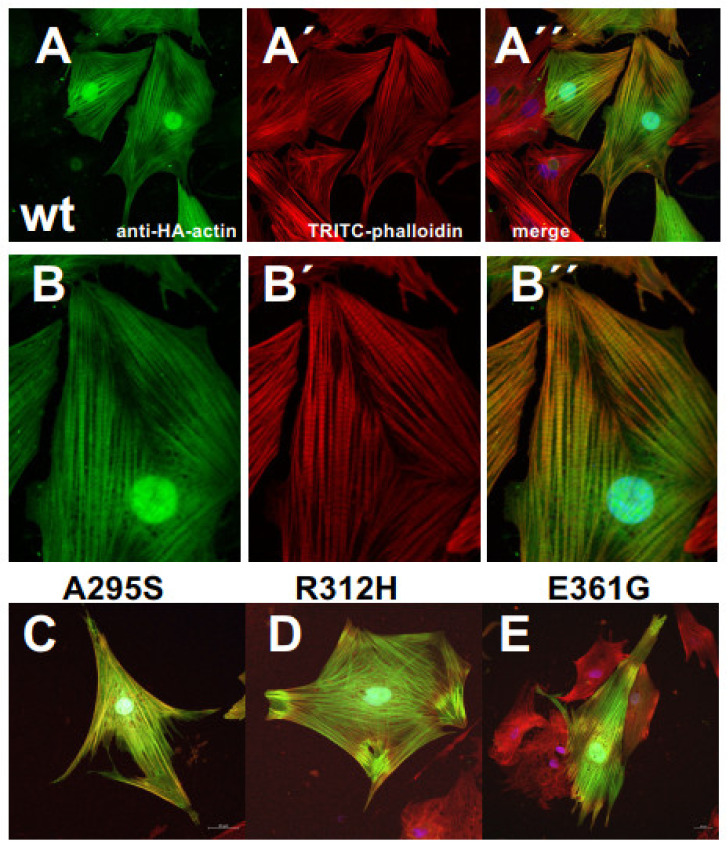
Neonatal rat cardiomyocytes (NRC) expressing HA-tagged c-actin variants. (**A**,**B**) Transduction of NRCs with adenoviral constructs encoding wt c-actin and subsequent immunostaining with anti-HA antibody (green), (**A’**,**B’**) TRITC-phalloidin (red), and (**A”**,**B”**) merged images also including nuclear staining with Hoechst 33342 (blue). (**B**–**B”**) Gives higher magnification of (**A**–**A”**) to visualize the cross-striation. (**C**–**E**) Merged images of NRCs expressing after transduction the p.A295S, R312H, and E361G mutants, respectively, stained by the identical procedure. Bars: 20 µm.

In order to observe specific effects of the cardiac α-actin variants in their natural cell type, adenoviral constructs of the c-α-actin variants were generated as described (see Materials and methods) and used to transduce neonatal rat cardiomyocytes (NRCs) isolated from one to three days old new-born rats. For these experiments, the c-α-actins were N-terminally HA-tagged for selective immunostaining. Subsequent TRITC-phalloidin staining allowed us to observe their incorporation into the intracellular filament system, in particular into nascent sarcomeres (Figure 6). The data indicate that the exogenous c-α-actins preferentially incorporated into existing or formed centrally located (around the nuclei) filamentous structures, whereas the TRITC-phalloidin staining appeared to be concentrated in peripheral filaments (Figure 6A–E). Higher magnification of NRCs infected with wt HA-c-α-actin showed a gradual increase in the concentration of the exogenous c-α-actin towards the cell center (Figure 6B), whereas the TRITC-phalloidin staining became weaker towards the cell center (Figure 6B) as indicated by a shift in the staining color from yellow (overlap region) to green (Figure 6B’). Sarcomeric structures were apparent in the periphery and within the yellow overlap region, whereas they appeared to vanish in the perinuclear region at the cell center (Figure 6B’), suggesting that the transfected wt c-α-actin was generated within the perinuclear region and initially formed microfilaments (Figure 6B) that appeared to become subsequently transformed into sarcomeric structures.

Only the p.R312H mutant showed an additional peripheral localization when HA-tagged (Figure 6D). The fact that the centrally located filamentous c-α-actins were only weakly stained by TRITC-phalloidin may suggest that the HA-tag of the exogenous c- α-actins impaired its binding.

**Figure 7:**
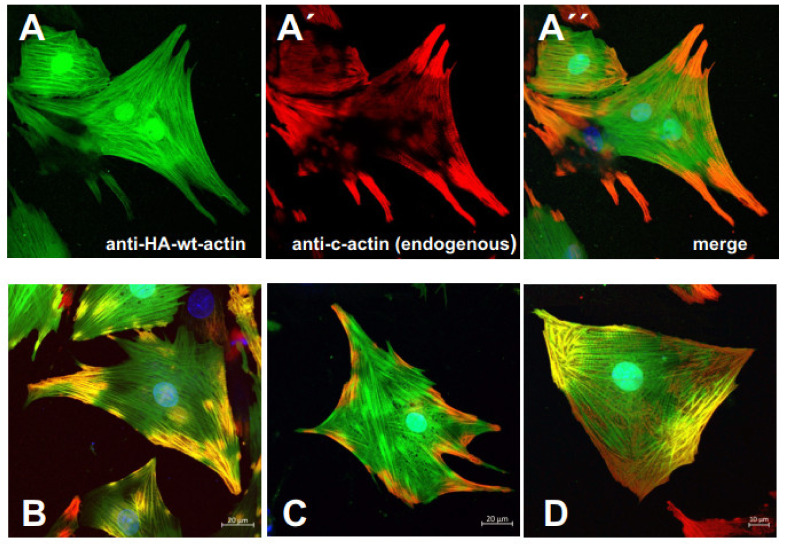
NRCs expressing HA-tagged c-actin variants counterstained with anti-c-actin mAB. (**A**) NRCs transduced with an adenoviral vector containing HA-tagged wt c-actin stained with anti-HA (green) and (**A’**) with anti-c-actin mAB (red), (**A”**) merge. Note localization of endogenous c-actin in cell periphery and extensions. (**B**) Merged images of identical immunostaining of NRCs infected with HA-tagged p.A295S; (**C**) with p.R312H, and (**D**) with p.E361G. Note co-localization of endo- and exogenous c-actin in (**B**,**D**), whereas in (**C**) NRCs were infected with HA-tagged p.R312H the endogenous c-actin appears more peripherally concentrated. Bars: 20 µm.

Since the N-terminal HA-tag blocks the binding of the monoclonal anti-cardiac α-actin antibody [27], it became possible to differentiate by immunostaining the localization of endogenous and exogenous c-α-actins. Indeed, the obtained data supported the assumption of a distinct distribution of endogenous and exogenous c-α-actins in infected NRCs. Figure 7 shows a clear concentration of the anti-c-α-actin mAB immunostaining for endogenous actin (red; Figure 7A and merge A”) at the cell periphery or in peripheral extensions of NRCs expressing wt HA-c-α-actin (anti-HA immunostained in green; Figure 7A). Similar results were obtained for NRCs infected with the p.A295S mutant (Figure 7B; only merged image), the p.R312H mutant (Figure 7C), or the p.E361G mutant (Figure 7D). In NRCs transduced with vectors coding for the p.A295S or p.E361G mutant, the endogenous c-α-actin was concentrated in peripheral extensions but also present underneath the plasma membrane along the whole cell periphery where it appeared to partially co-localize with the exogenous mutant α-actins (Figure 7B,D). Nevertheless, the exogenous c-α-actins were clearly concentrated in the middle of the infected NRCs.

**Figure 8:**
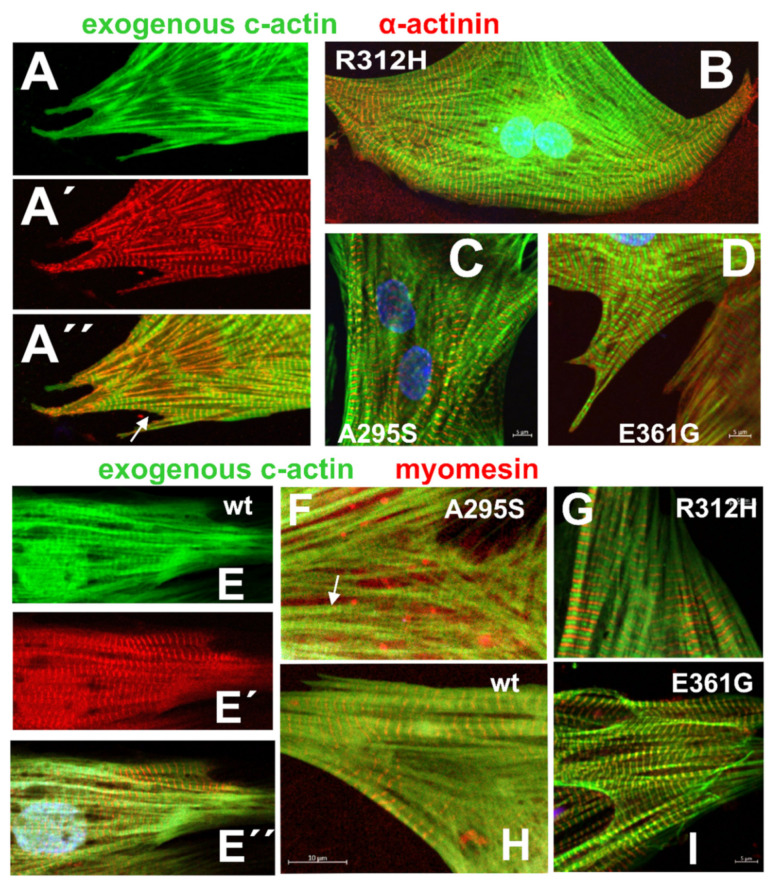
NRCs expressing HA-tagged c-actin variants counterstained with anti-α-actinin or anti-myomesin. (**A**) NRC transduced with vectors coding wt HA-tagged c-actin stained with anti-HA (green) were counterstained with (**A’**) anti-α-actinin (red) showing cross-striations (arrows). (**A’’**) gives the merged image. Note that cross-striation appears in many cases yellowish stained indicating co-localization (arrows). (**B**–**D**) gives merged images of identical staining protocol for p.R312H (**B**), p.A295S (**C**), and p.E361G (**D**). Note that in (**B**,**C**), the cross-striations are yellowish stained indicating also for HA-p.R312H and HA-p.A295S co-localization with α-actinin suggesting preferred incorporation in the region of the Z-line, i.e., at the plus-ends. In contrast, HA-p.E361G (**D**) transduced NRCs show a clear separation of the anti-HA (green) and anti-α-actinin immunostaining (red), indicating its incorporation at the minus ends in the region of the M-line. (**E**–**E’’**) Similiar immunostaining of wt HA-tagged c-actin (green) and anti-myomesin (red). Note the separation of anti-HA and -myomesin in (**E”**,**H**). (**F**) Merged image for HA-p.A295S and (**G**) for HA-p.R312H c-actins. Note the separation of anti-HA and -myomesin in (**F**,**G**), indicating incorporation of the minus ends in the region of the M-line. In contrast, (**I**) NRCs transduced with HA-p.E361G indicate co-localization of this c-actin mutant with myomesin supporting the notion that its incorporation into sarcomeric structures occurs at the minus ends of existing actin filaments in the region of the M-line. Bars give 20 µm.

Finally, transduced NRCs were counterstained with anti-α-actinin or -myomesin to identify the site of incorporation of the exogenous c-α-actins. Three days after isolation, the NRCs had formed intracellular sarcomeric structures, which in some instances spanned the whole cytoplasm. Immunostaining with anti-α-actinin (Figure 8A–D) or anti-myomesin (Figure 8E–I) identified the Z- or M-lines, to which the thin filaments are attached by their plus ends or mark the minus ends, respectively. The data obtained showed that only the p.E361G actin was preferentially incorporated at the minus ends (co-localization with anti-myomesin staining in Figure 8I), whereas wt, p.A295S, and p.R312H appeared to incorporate preferentially at the plus ends, i.e., at the region of thZ-lines (co-localization with anti-α-actinin staining in Figure 8A’,B,C). The preferential incorporation of the p.E361G actin at the minus end might be due to the fact that the mutated site is within the binding region of α-actinin, a Z-line component. These data suggest that in case of a high expression level of the p.E361G DCM mutant, the thin filament attachment to Z-line might be impaired, leading to reduced stability of the sarcomeres.

## 4. Discussion

Hypertrophic and dilative cardiomyopathies (CM) are the most frequent genetic diseases of the heart, and a high percentage are caused by point mutations of genes coding for sarcomeric proteins. Missense mutations of cardiac actin have been identified as a cause of hypertrophic or dilated cardiomyopathy, apparently depending on the localization of the mutation within the molecule [5,17].

Here we investigated whether the interaction of c-actin mutants with supposedly non-sarcomeric proteins was also altered and correlated with the type of CM induced. For this study, we employed wild-type cardiac α-actin, the p.A295S mutant causing HCM, and the DCM causing p.R312H and p.E361G mutants. Previous investigations on the interaction of these c-actin variants with other sarcomeric proteins have shown that the HCM p.A295S mutant possesses an increased Ca^2+^-sensitivity and the DCM p.R312H and p.E361G mutants a decreased Ca^2+^-sensitivity leading to hyper- or hypocontractlity and subsequently to the induction of HCM or DCM, respectively [17].

After purification, the cardiac α-actins were pure as judged by SDS-PAGE and have been shown to maintain their native state [7,27]. All recombinant produced c-actins polymerized with comparable rates (Figure 2 and Table 1), and their rate of polymerization was stimulated by the Arp2/3 complex and the FH2-domain of the formin mDia3. These actin polymerization nucleators are non-sarcomeric proteins though the presence of formins has been shown in adult cardiomyocytes. They may, however, be involved in the organization of the initial microfilament systems during cardiomyocyte development [39].

Recently detected interaction partners of actin are flavin proteins with monooxygenase activity of the MICAL family. MICAL-1 modifies filamentous actin by covalent oxidation of two methionines located in subdomain 2 (residues 44 and 47) and thereby induces its de-polymerization [23,24,25]. The monooxygenase of MICAL is located in the N-terminal domain that in non-stimulated cells is auto-inhibited by interacting with its C-terminal part [25,30]. Cell stimulation by external signals leads to a relief of the auto-inhibited monooxygenase activity and subsequently to rapid F-actin de-polymerization. Active MICAL-1 is present in the contractile ring of cells during cytokinesis [30] and executes repulsive signals to growing neurites leading to arrest of their migratory activity [23,24,25]. MICAL proteins have also been implicated in Drosophila myocyte development [28]. Here we were able to demonstrate the presence of MICAL-1 in ARCs in close proximity to actin (Figure 3D). In addition, we investigated the rate of oxidation and de-polymerization of the c-actin variants by MICAL-1. Indeed, observed c-actin variant-specific differences in the rates of oxidation and de-polymerization (Figure 3A,B). Since none of the mutations were localized close to the affected methionines in subdomain 2, we have to assume that the mutations led to conformational alterations of most likely subdomain 2, which is known to be a rather flexible part of actin [40]. Surprisingly, recombinantly expressed wt c-actin exhibited lower rates of oxidation and de-polymerization than bovine c-actin and the p.A295S mutant. This difference can presently not be explained. Nevertheless, the high rates for the p.A295S mutant agree with the reported high rate of turnover of this mutant in insect muscles [41]. The considerably decreased rates of MICAL induced de-polymerization of both DCM mutants (p.R312H and p.E361G) might point to increased persistence of these mutants in sarcomeric structures and be of consequences during cardiomyocyte development and differentiation and contribute to DCM induction.

In the past, the effects of actin oxidation after exposure to, for instance, H_2_O_2_ have been investigated under the assumption that alterations in the cellular response to oxidative stress are mediated by direct modification of the actin cytoskeleton [42,43]. These studies used either skeletal muscle of cytoplasmic ß-actin and indeed showed specific oxidation of the cysteines 374 or 272, leading to de-polymerization and/or reduced polymerizability. Oxidation of Cys272 also led to the inhibition of ß-actin to interact with profilin [37]. In contrast to actin oxidation by reactive oxygen species (ROS), MICAL-1 acts as a reversible signaling effector independent of the redox state of the cellular environment.

In addition, we investigated the behavior of the c-actin mutants in a cellular context and therefore transfected the c-actin variants into Hela and the MDCK cell line (Figure 4 and Figure 5) or rat neonatal cardiomyocytes (Figure 6 and Figure 7). Transfection of non-muscle cell lines indicated incorporation of all c-α-actins into the cellular cytoskeleton. Wt and p.A295S c-α-actins were preferentially incorporated into the microfilament systems, whereas the p.R312H and p.E361G mutants incorporated into the submembranous cortical F-actin network, more typical of cytoplasmic actins. This behavior was particularly evident in MDCK cells and might appear surprising since the six mammalian actins contain identical residues at positions 295, 312, and 361 (A, R, and E, respectively). Therefore, the mutations at positions 312 and 361 might have reduced the strength of their interactions with filament-forming actin-binding proteins. This might especially be the case for the p.E361G mutant, whose rate of polymerization was not stimulated by mDia3-FH2.

Next, we performed transduction experiments of neonatal rat cardiomyocytes (NRCs). The c-α-actin variants were found to incorporate into microfilamentous or sarcomeric structures and at least partially co-polymerized with endogenous c-actin (Figure 7 and Figure 8). A clear different intracellular localization of exogenous and endogenous c-actins was found in one to two-day-old NRCs. These cells showed a more central localization of the exogenous actins in sarcomeric and microfilamentous organizations, irrespective of the variant. In contrast, the endogenous c-α-actin was concentrated in a more peripheral microfilamentous and sarcomeric form (Figure 7A’). Sarcomerogenesis has been shown to start at the cell periphery at sites of attachment to the extracellular matrix and to subsequently extend centripetally [18]. These sites (also termed proto-costameres) possibly developed before translation of the adenovirally introduced exogenous c-α-actin variants and were therefore formed by endogenous c-α-actins. Therefore, the adenovirally introduced exogenous c-actins incorporated preferentially into central sarcomeric structures. However, it is also possible that the mRNA of endogenous c-α-actin contained additional localization signals that directed its translation to the cell periphery that are absent in the adenoviral constructs [44].

Finally, we determined the site of incorporation of the exogenous c-α-actins into the sarcomeric thin filaments by co-staining with anti-α-actinin and -myomesin, markers of the Z- and M-line, respectively. All c-α-actins appeared to incorporate at the Z-line, i.e., the thin filament plus end except the p.E361G mutant, which preferentially incorporated in the region of the M-line. The p.E361G residue is localized in subdomain 1 of actin and has been implicated in binding to α-actinin [18]. Indeed, it has been shown that the p.E361G mutation possesses an about threefold lower affinity to α-actinin [45]. Furthermore, the p.E361G appears to have a high affinity for minus ends, as indicated by its fast rate of polymerization. These properties will impair (Figure 8D) but not completely exclude incorporation at the Z-line. Indeed, co-localization with myomesin indicated weak immunofluorescence between M-lines (Figure 8I), suggesting incorporation albeit reduced at the Z-line. Under conditions of mechanical stress, the reduced affinity of the p.E361G mutant to the Z-line component α-actinin might lead to sarcomere disruptions, finally causing DCM.

Our data show that recombinant wt c-α-actin behaved similarly to conventionally purified bovine c-α-actin except for its lower rate of oxidation by MICAL-1. Nevertheless, this concordance strengthens the assumption that the isolated mutant c-α-actins were also in their native state and exhibited their intrinsic functionality. In summary, our data indicate that each c-actin mutation possesses specific differences to wt c-actin in the mode of interactions with other binding proteins. In a different investigation, we demonstrated that the HCM p.A295S mutant possesses a higher and the DCM p.R312H and p.E361G mutants a lower Ca^2+^-sensitivity, respectively [7]. This conclusion is strengthened by our transfection data, which suggest for both DCM mutants preferential incorporation into cellular F-actin networks of Hela and MDCK cells and the p.E361G mutant incorporation both thin filament ends of NRCs. These alterations can lead to differences in the mechanical stability of the cardiomyocyte thin filaments.

## 5. Conclusions

After expression of cardiac actin and its HCM mutant p.A295S and DCM p.R312H and p.E361G mutants by the baculovirus/Sf21 insect cell system and their subsequent purification, we investigated first their polymerizability and subsequently determined the effect of the Arp2/3 complex and mDia-FH2 domain on their rate of polymerization. The data obtained indicated variant-specific differences. In addition, we showed that the monooxygenase MICAL-1 is able to specifically oxidize all c-actin variants, albeit with different rates also leading to differences in their oxidation-induced rates of de-polymerization. This is a novel test for the functionality of the c-actin and c-actin mutants that may be related to differences in their incorporation and persistence in sarcomeric structures. Indeed, differences in their incorporation into distinct supramolecular organizations of F-actin became apparent by transfection experiments into established cell lines. Infection of neonatal rat cardiomyocytes showed their incorporation into the plus ends of the sarcomeric thin filaments except for the p.E361G mutant, which incorporated preferentially at their minus ends.

## Figures and Tables

**Table 1:** Effect of polymerization nucleators on cardiac actin variants. Polymerization parameters of 5 µM cardiac actin variants in the absence or presence of 50 nM Arp2/3 complex or 50 nM mDia3-FH2 (data taken from Figure 3D–G). The experiments were repeated three times with two different protein preparations with no significant differences.

Half Times of Polymerization (t_1/2_) in Minutes in the Presence of Nucleators (Data Taken from Figure 3D–G)				
	Recomb. wt c-actin	p.A295S	p.R312H	p.E361G
**Control**	8.6	12.2	13.8	4.0
**Arp2/3 complex**	2.1	1.2	1.0	1.8
**mDia3-FH_2_**	6.9	3.5	4.1	2.9

## Data Availability

Data are deposited and stored in mobile and hard discs of institutional computers (Institut für Forschung und Lehre (IFL), Molecular and Experimental Cardiology, Ruhr University Bochum, 44801 Bochum, Germany).
